# 

**Published:** 1859-09

**Authors:** 


### CONTENTS.

Use of Collodion in exposure of Nerves.—By ABR. ROBERTSON...... 101
Experience with Atmospheric Plates. By A. BERRY, D.D.S...... 104
Amalgam Work,...... 108
Impressions,...... 110
Dental Puffing,...... 111
Pressure Plate. BY J. TAFT...... 112
A Filtering Blow-Pipe...... 114
Remarks on Porcelain Work. By M. LOOMIS,...... 115
Fang Filling. By W. H. SHADOAN...... 117
Proceedings of Societies,...... 120
Editorial,...... 147

